# Nontraditional Use of TEE to Evaluate Hepatic Vasculature and Guide Surgical Management in Orthotopic Liver Transplantation

**DOI:** 10.1155/2019/5293069

**Published:** 2019-06-19

**Authors:** Narjeet Khurmi, Mitchell Seman, Brantley Gaitan, Scott Young, David Rosenfeld, Emmanouil Giorgakis, Winston Hewitt, Amit Mathur

**Affiliations:** ^1^Department of Anesthesiology and Perioperative Medicine, Mayo Clinic Arizona, Phoenix, AZ 85054, USA; ^2^Department of Radiology, Mayo Clinic Arizona, Phoenix, AZ 85054, USA; ^3^Department of Surgery, Division of Transplant Surgery, Mayo Clinic Arizona, Phoenix, AZ 85054, USA

## Abstract

Intraoperative Transesophageal Echocardiography (TEE) during orthotopic liver transplant (OLT) is used to gather real-time information on cardiovascular function and intravascular volume status. We report a case where nonstandard TEE views were used to inspect the hepatic vasculature after allograft implantation. A 29-year-old male with secondary biliary cirrhosis with a MELD score of 20 underwent OLT using a liver from a 21-year-old brain-dead donor. Postreperfusion TEE, using the modified hepatic vein views, confirmed the presence of an inferior vena cava (IVC) suprahepatic anastomotic stenosis and hepatic vein and IVC thrombus resulting in hepatic venous outflow obstruction, allograft congestion, and hemodynamic instability. These nonstandard TEE images established the extent of suprahepatic caval outflow obstruction, in which intraoperative ultrasound was unable to definitively demonstrate. This guided real-time surgical decision-making in the postimplantation phase of the operation—ultimately leading to hepatic vein and IVC thrombectomy and revision of suprahepatic caval anastomosis.

## 1. Introduction/Background

Intraoperative Transesophageal Echocardiography (TEE) has continued to expand over the last 20 years. Although TEE is most often used during cardiac surgery, TEE use in liver transplantation has grown significantly [1]. Routine use of TEE during orthotopic liver transplant (OLT) is reported to be 40-72% in high-volume liver transplant centers [2, 3]. The decision for elective TEE examination should be guided by the extent of surgical procedure, the patient's medical status, and the anticipated perioperative physiologic changes.

Advances in medical imaging have enhanced diagnostic accuracy resulting in more effective medical interventions. Echocardiography utilizes ultrasound waveforms to visualize cardiac structures and provides real-time information on active physiologic processes [4, 6]. TEE is relatively noninvasive, and when compared to Transthoracic Echocardiography (TTE), TEE allows for better visualization of posterior cardiac structures and is often more practical for intraoperative monitoring, as it avoids contamination of the surgical field. The American Society of Echocardiography and Society of Cardiac Anesthesiologists have consensus guidelines for both the Basic and Comprehensive TEE Exams.

Basic intraoperative TEE during OLT is primarily used to gather real-time information on left ventricular size and function, valvular anatomy and function, volume status, and pericardial abnormalities [4]. In addition, TEE can be used to diagnose and assess for intracardiac thrombus, left ventricular outflow tract obstruction, right heart function, pulmonary embolus, pulmonary hypertension, cardiac shunts, pericardial and cardiac tamponade, aortic atheromas, and acute aortic syndromes [6–9].

**Figure 1:**
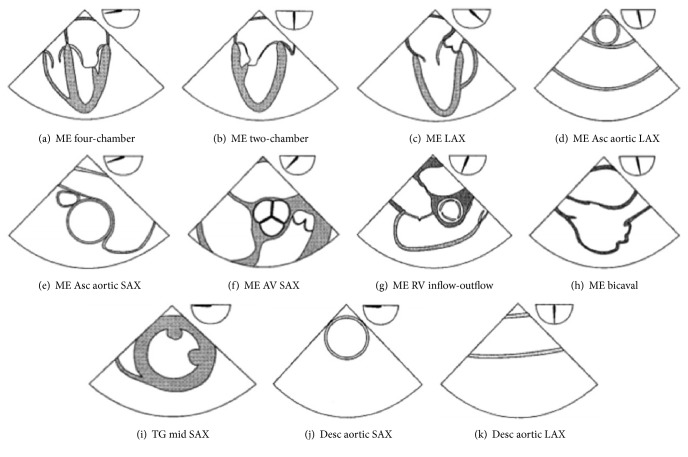
Cross-sectional views of the 11 views of the ASE and SCA basic perioperative TEE examination. Multiplane angle is indicated by the icon at the top right of each view [4].

The Basic TEE Exam includes 11 primary views (Figure 1): Mid-esophageal (ME) four-chamber, ME two-chamber, ME long-axis (LAX), ME ascending aortic LAX, ME ascending aortic short-axis (SAX), ME Aortic Valve SAX, ME Right Ventricular Inflow-Outflow, ME Bicaval, Transgastric mid SAX, Descending aortic SAX, and Descending aortic LAX. The Basic TEE exam is intended to be nondiagnostic and excludes assessments that modify the surgical plan, as those assessments require advanced perioperative TEE skills [4].

As its name implies, the Comprehensive (Advanced) TEE examination includes additional 17 views when compared with the Basic TEE exam for a total of 28 views. This gives the clinician a much more detailed evaluation of cardiac physiology and is often employed during complex cardiac cases to evaluate real-time physiologic changes that occur during surgery. These more comprehensive views can lead to information that has the potential to actively change the surgical plan. Thus, the comprehensive TEE exam is intended to have diagnostic utility along with the ability to guide perioperative decision-making [4].

A recent case series by Vetrugno et al. described that TEE is not just limited to cardiac evaluation in patients undergoing orthotopic liver transplantation (OLT) but can be used to evaluate other organs including liver, lung, spleen, and kidneys [10]. In this report we will describe a case at our institution in which nontraditional TEE imaging was used to diagnose intraoperative noncardiac pathology, which lead to definitive surgical care.

## 2. Case Presentation

A 29-year-old male with end-stage liver disease due to secondary biliary cirrhosis with a MELD score of 20 presented for orthotopic liver transplant. His liver disease was complicated by portal hypertension, hepatic encephalopathy, jaundice, and pruritus. Additionally, he had an asymptomatic holosystolic cardiac murmur. The patient received a postcross clamp offer from a 21-year-old brain-dead donor with apparent 40-50% fat. Intraoperative monitoring included an Arterial line, CVP monitoring, and TEE.

The operation was performed with caval replacement, portal vein (PV) to PV, and recipient hepatic artery (HA) to reconstructed donor HA. Prior to reperfusion the patient was on 0.04mcg/mg/min of epinephrine and had required 3 units of packed red blood cells (PRBCs). At reperfusion the patient received multiple boluses of 2-4 units of vasopressin and 25-50mcg boluses of epinephrine. An hour following the initial hypotension at reperfusion, the patient persisted with hemodynamic instability, requiring 5 units of PRBCs, multiple fluid boluses, and the addition of 0.04 units/minute of vasopressin, 1mcg/kg/min of phenylephrine, and 0.1mcg/kg/min of norepinephrine. At this time the allograft appeared congested and enlarged.

**Figure 2:**
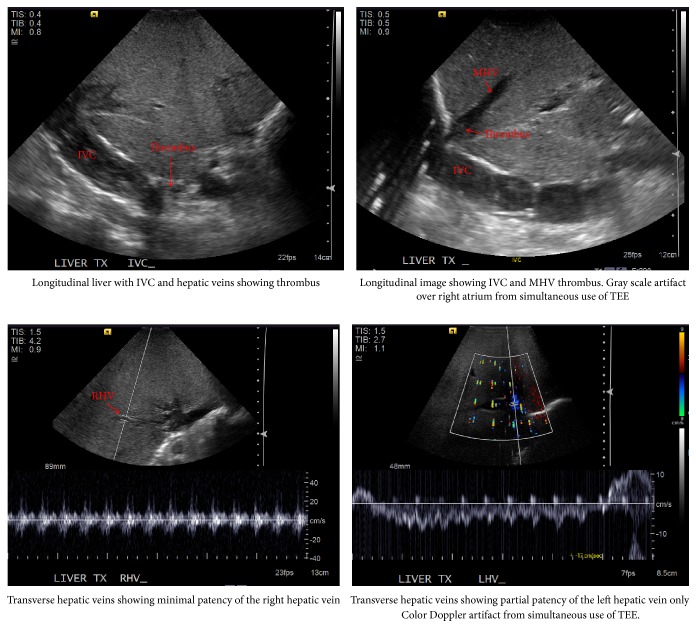
Intraoperative US images demonstrating caval obstruction. Inferior vena cava (IVC), middle hepatic vein (MHV).

Intraoperative ultrasound (US) was used by the surgeon and radiologist to evaluate the intrahepatic vessels for flow (Figure 2). Initially, thrombus was noted in the hepatic veins, then in the IVC. The window to visualize extension above the liver was not possible due to lung and intra-abdominal gas. The liver exposure was not enough to place a probe directly on the hepatic vein/IVC junction. Therefore, the cause and extent of the suprahepatic caval obstruction was incompletely visible.

**Figure 3:**
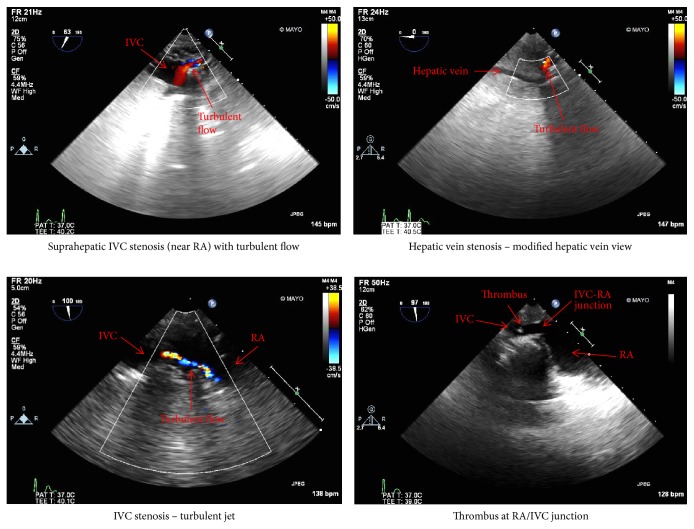
Showing intraoperative TEE images demonstrating IVC suprahepatic anastamotic stenosis and hepatic vein thrombosis.

**Figure 4:**
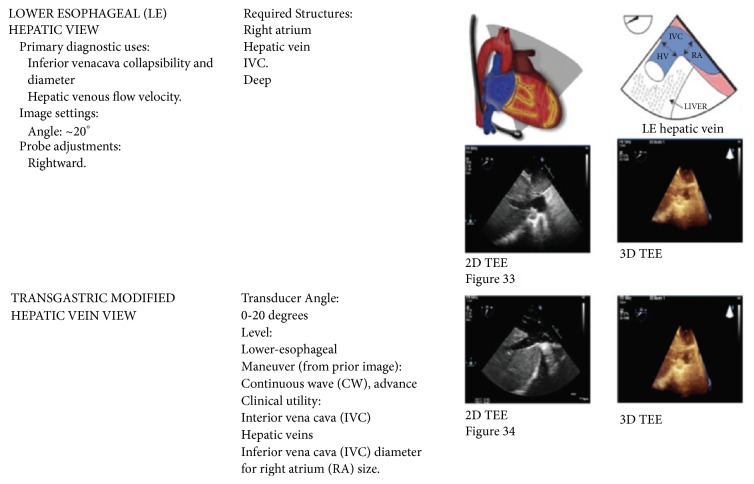
Modified hepatic views with corresponding anatomical model [5].

Intraoperative TEE examination was performed to more thoroughly assess the cause and extent of the thrombus. Modified transgastric hepatic vein view (Figure 4) was obtained first by rotating the probe clockwise from the transgastric view, and then opening the omniplane angle to about 60 degrees to find the long-axis view of the hepatic vein. Alternating between the modified transgastric hepatic vein view and a modified bicaval view, we were able to view the IVC from the hepatic vein to the atriocaval junction. This confirmed the presence of an inferior vena cava (IVC) suprahepatic anastomotic stenosis and hepatic vein thrombus resulting in hepatic outflow obstruction, allograft congestion, and hemodynamic instability (Figure 3).

The echocardiographic findings guided real-time surgical decision-making in the postimplantation phase of the operation, ultimately leading to IVC thrombectomy and revision of suprahepatic caval anastomosis which resulted in subsequent allograft decompression. The patient recovered from OLT and has normal graft function at 18 months postoperatively.

## 3. Discussion

Intraoperative TEE exam is increasing and advocated in OLT, as recipient patients are typically sick with many serious medical comorbidities [7]. Nontraditional use of TEE to diagnose intraoperative noncardiac pathology in OLT appears underreported. Intraoperative TEE is a valuable tool to assess hepatic vascular structures critical to allograft function and survival without interruption of the surgical procedure. Of particular use are the modified views including the lower esophageal hepatic and transgastric hepatic vein views (Figure 4). Anatomic proximity of the distal esophagus and gastric antrum allow for adequate visualization of hepatic veins and detection of hepatic vein flow by TEE.

Despite the increasing prevalence of TEE use during liver transplantation, Basic TEE certification is not a requirement to perform anesthesia for OLT. In one study, only 64% of responding centers had at least 1 anesthesiologist with Basic TEE certification [1]. At low-volume transplant centers, liver transplant anesthesiologists may have difficulty maintaining Basic TEE certification due to the minimum requirement of 25 TEE exams/year. One option would be to actively use TEE in major abdominal or vascular cases, to offset inadequate TEE exams during OLT. The modified hepatic vein views described in our case are not part of the Basic TEE certification endorsed by American Society of Echocardiography and Society of Cardiovascular Anesthesiologists. With this in mind, further research is needed to standardize these described modified views with the goal of improving their diagnostic accuracy and utility in OLT. In our case report, nonstandard TEE images were obtained using the modified transgastric hepatic vein view and lower esophageal hepatic vein view to diagnose and guide real-time surgical management ultimately leading to graft preservation and patient survival.

## Data Availability

Data sharing is not applicable to this article as no new data were created or analyzed in this study.

## Abbreviations

TEE: Transesophageal echocardiography
OLT: Orthotopic liver transplant
IVC: Inferior vena cava
PV: Portal vein
PRBC: Packed red blood cells
HA: hepatic artery
US: Ultrasound.
